# Advancing carbon estimation in harvested wood products in the United States: a case study in the Northern Lake States

**DOI:** 10.1186/s13021-026-00464-y

**Published:** 2026-06-08

**Authors:** Joseph M. Nash, Grant M. Domke

**Affiliations:** 1 USDA Forest Service, Northern Research Station, Forest Inventory and Analysis Program, Saint Paul, MN USA; 2 Oak Ridge Associated Universities, Oak Ridge, TN USA

**Keywords:** Harvested wood products, forest inventory and analysis, Nationwide Forest Inventory, Carbon

## Abstract

**Background:**

Carbon stocks and stock changes in harvested wood products (HWPs) are an important part of land sector greenhouse gas (GHG) estimation and reporting. HWPs broadly categorized as products in-use (e.g., solid wood and paper products) and in solid waste disposal sites (SWDS; e.g., landfills), store carbon transferred from harvested trees. In the United States (US), estimates of carbon in HWPs have historically been reported in the US GHG Inventory and included in submissions to the United Nations Framework Convention on Climate Change. These data have been obtained from national and international statistics on production and consumption of forest products and incorporated into a compilation system to estimate carbon in products in-use and in SWDS. In contrast, estimates of carbon in forest ecosystems have been obtained from nationwide forest inventory (NFI) data collected and maintained by the US Forest Service, Forest Inventory and Analysis (FIA) program. Here we describe a case study for the Northern Lake States region of the US (Michigan, Minnesota, Wisconsin) where harvest data from the FIA program were integrated into HWP compilation systems. This advance improves consistency and continuity with forest ecosystem from NFI plots with estimates of HWPs.

**Results:**

Over the 1900–2024 time period, total estimated net accumulation (i.e., balance of additions from transfers of harvested wood from forest ecosystems and losses from decay of wood harvested in the past) of carbon stored in products in-use was 277.0 ± 17.5 Million Metric Tons (MMT) Carbon (C) and in SWDS was 155.2 ± 9.8 MMT C. We estimate that HWPs from the region represent a carbon sink of 4.9 ± 0.1 MMT C in 2024. These estimates include HWPs produced in the region and exported domestically or internationally, as well as any HWPs produced and retained in the region, but not imports.

**Conclusions:**

The proposed methodology enables disaggregation with coarse national and state-level FIA data, and allows for integration of more specific, entity-level data to improve precision and reduce uncertainty in HWP estimates in the US and improves consistency and continuity with forest ecosystem estimates across spatial and temporal scales.

**Supplementary Information:**

The online version contains supplementary material available at 10.1186/s13021-026-00464-y.

## Background

Forest ecosystems represent the largest terrestrial carbon sink on Earth [61]. As living trees photosynthesize and grow, they transfer carbon dioxide (CO_2_) from the atmosphere and store carbon in the physical structure of their biomass. In addition to forest ecosystems sequestering and storing carbon, wood harvested from these ecosystems and stored in harvested wood products (HWPs) may contribute to climate change mitigation [8, 71]. In the United States (US) in 2023, CO_2_ transfers from the atmosphere and other land use categories by forest ecosystems and harvested wood products (HWPs) represented an offset equivalent to approximately 16.1% of total domestic greenhouse gas emissions, about 1.5% of which is accounted for by HWPs [16]. As a commitment to reducing the concentration of GHGs in the atmosphere, 198 countries are party to the 1992 United Nations Framework Convention on Climate Change (UNFCCC) and report on carbon stocks and flux within forest ecosystems using methodological guidance from the Intergovernmental Panel on Climate Change (IPCC) [72]. There are five ecosystem carbon pools (aboveground biomass, belowground biomass, dead wood, litter, and soil) as well as the harvested wood products (HWPs) pool which is separated into products in-use and in solid waste disposal sites (SWDS). The HWPs pool represents transfers of carbon from ecosystem pools (aboveground biomass and dead wood) into distinct HWPs categories with varying longevity as trees are harvested, transported, milled into products, placed into various end uses, and eventually discarded. In the forest sector, when aboveground biomass and/or dead wood are harvested, some of that carbon may be transferred from the ecosystem to HWPs with a range of short-term to long-term lifespans. In the US forest carbon estimation and reporting system, these transfers are presented as a loss from the forest ecosystem and a gain in the HWPs category [14, 16].

In addition to national reporting in the US, state agencies often report estimates of carbon stocks and stock changes from forest ecosystem carbon pools and HWPs as commitments to sustainable forest management and to decreasing GHG emissions [37, 45, 84]. Estimates of total carbon stored in forest ecosystem pools are also used in voluntary and regulatory carbon markets, where fossil fuel emitters purchase “credits” from owners and managers of forest ecosystems that are actively removing CO_2_ from the atmosphere and storing it as biogenic carbon [1, 9, 17, 17, 19, 19, 20]. Therefore, there is a need for compilation systems that can provide estimates of carbon transferred from forest ecosystems to HWPs across spatial and temporal scales.

Aside from instances of combustion, carbon stored in trees is not released to the atmosphere immediately when a tree is harvested. In the US, emissions associated with harvesting, transportation, and processing are accounted for in other sectors (e.g., manufacturing) and thus are not included in HWP carbon estimation. For trees that are processed into HWPs, the carbon stored within the structure of the wood will be stored for as long as the product stays in-use, and unless the product is burned, the carbon will remain stored for years after it has been discarded due to the slow rates of decay in SWDS [36]. There are several methodological approaches that the IPCC [31] includes in guidelines for HWP carbon estimation. The US has historically used the production method for UNFCCC reporting, which accounts for all HWPs produced in the US and exported outside of the US, but does not include imports [16]. Transparency of the estimation approach used is important, particularly on a global scale, to avoid double-counting or omissions in the HWPs category.

The forest ecosystem carbon estimation guidance presented by the IPCC [31] follows a tiered approach that corresponds to varying levels of data availability and complexity; Tier 1 methods provide broad default parameters for use when no regional data are available, Tier 2 methods are available for entities that have limited regional data, and Tier 3 methods use specific parameters from regional studies. The precision and accuracy of estimates can be improved by moving toward Tier 3 methodologies through the integration of regional parameters in an estimation framework [31]. For example, the IPCC guidelines provide a default parameter for the amount of carbon in wood, but species-specific carbon fractions are available for the US [81].

At a national level in the US, carbon in HWPs has historically been estimated using a compilation process in an application referred to as WOODCARBII [62, 87]. This compilation process relies on data from various survey and reporting metrics on HWPs produced in, imported to, and exported from the US, including the USDA Forest Service forest inventory and analysis (FIA) Program timber products output (TPO), the Food and Agriculture Organization (FAO), and various timber product associations (see appendix table A-198 in [16]). Although reports often cite estimates from 1990, the system estimates HWPs from 1900 to include carbon that is still present in HWP in-use and in SWDS from historical harvests. The compilation process relies on several conversion factors to estimate the amount of carbon in HWPs as the statistics from reporting entities are typically in industry standard units (e.g., cubic feet, oven-dry tons). To estimate carbon from harvested trees in the US using the existing compilation process, estimates of HWPs produced by the US (minus imports) were back calculated to the harvest estimates required to produce those products. Estimates of tree harvests calculated from HWP estimates in the compilation process, however, do not incorporate harvest removals from NFI plots and assume a default ratio of 50% carbon in all wood. Furthermore, within the existing framework there is no way to disaggregate estimates from the national level for state and entity-level reporting. That said, the existing compilation process has been adapted to disaggregate estimates to the state or entity-level [11, 30, 34, 48, 49, 69, 70] but these adaptations do not currently incorporate NFI harvest removals. Since forest ecosystem estimates are obtained from NFI data, integrating harvest information from NFI plots would improve consistency and continuity between estimates of carbon in HWPs and forest ecosystem pools. Further, a compilation system that accommodates data across scales would facilitate disaggregation of HWP estimates from the nation to the entity, improving consistency and continuity in reporting in the US.

Estimating the amount of HWPs in-use and in SWDS requires assumptions and conversions regardless of the methodology. Even with a robust field inventory, annualized estimates of harvested trees at an entity, state, or national scale come with different levels of uncertainty dependent on the scale of the analysis – particularly for historical harvest estimates made before robust forest inventory data were available. Therefore, any attempt to estimate harvested carbon, and the accumulated HWPs produced from those harvests, must include a measure of the uncertainty for estimates and associated assumptions to quantify the reliability of data used in the analysis, provide context for results, enable decision-making, and inform future investments in data collection and analysis.

Here, we provide proof of concept for a new compilation system, the US HWP CCS, which was built around NFI data to estimate carbon in HWPs in-use and in SWDS, both by the amount, species, and size of trees harvested, and in the amount of carbon in those trees. We also designed a system that eliminates assumptions related to carbon fractions and conversions from the amount of timber products to various primary products. We aimed to create a compilation system that can be used with limited data (Tier 1 or Tier 2), while also allowing for the incorporation of specific regional data, when available (Tier 3). Furthermore, we provide a methodology to disaggregate estimates to the state or entity level and account for the variability and uncertainty in contemporary and historical harvest estimates while accounting for uncertainties in HWP estimates and assumptions and variability in harvest records.

## Methods

### Harvested wood product accounting approaches

The IPCC [31] provides four accounting approaches to estimate carbon in HWPs, ’stock-change’, ‘production’, ‘atmospheric flow’, and ‘simple-decay’. The US has historically included the stock-change, production, and atmospheric flow approaches in the greenhouse gas inventories but used the production approach for reporting to the UNFCCC [16]. The choice of approach is important, particularly at a global scale to avoid double-counting or omissions due to the system boundaries and included parameters. The stock-change approach estimates the change in stock of HWPs within the country where the wood is consumed, including imports and excluding exports. The production approach estimates changes in stocks of HWPs produced from that country, including exported wood but not imports. In contrast, the atmospheric flow and simple-decay approaches are flux-based, i.e. they include estimates of fluxes of CO_2_ from and to the atmosphere from HWPs. The atmospheric flow approach differs from the simple-decay in that the former includes fluxes occurring within the country where the wood is consumed, including imports and excluding exports, and the latter includes all fluxes from wood produced from a country including exports and excluding imports. The results from this case study will be presented following the production approach but the US HWP CCS is set up to allow estimation from any of these approaches.

### Forest inventory and analysis

The FIA program (https://research.fs.usda.gov/programs/fia) includes NFI, National Resource Use Monitoring (NRUM), National Woodland Owners Survey (NWOS), and the Urban Inventory. The US HWP CCS was specifically built around NFI data and TPO information from NRUM to improve consistency and continuity with forest ecosystem estimates obtained from NFI data and to facilitate disaggregation from the nation to the entity. The NFI in the US was established in the late 1920s and has evolved from a state-by-state periodic inventory where forest land was sampled every 10–15 years in each state into a nationally consistent, annual inventory [2]. The transition from periodic inventories to the annual design began in the late 1990s. The annual NFI is a probabilistic sample with permanent base-intensity sample plots distributed approximately every 2,428 ha across all ownerships and land uses in the conterminous United States (CONUS) [2]. Field crews visit NFI plots with at least one forest land condition (i.e., a mapped domain on subplots) approximately every 5–7 years in the eastern US and every 10 years in the western US [80]. Each forested NFI plot is comprised of four fixed-radius subplots (7.32 m) spaced 36.6 m apart in a triangular arrangement with one subplot in the center. Tree- (e.g., diameter, height, status [live, dead, removed]) and site-level attributes are measured on all forest land conditions. The TPO function within the NRUM area of the FIA program has also evolved over time, from periodic to annual mill surveys, designed to determine the amount of harvest removals that are processed into primary wood products. TPO data are now collected annually, nationwide, with a mill survey using a modified stratified random sample of active primary wood processing facilities [12]. The NFI data were used to obtain harvest estimates and TPO data were used to constrain those harvest estimates and determine the proportion of harvests to allocate to various primary products, as well as determine the movement of those products.

### Geographic area

This analysis was focused on carbon in HWPs in-use and in SWDS from 1900–2024 in the Northern Lake States (NLS); Michigan, Minnesota, and Wisconsin, US. The system boundaries consisted of the geographical extent of the three NLS. Wood harvested from forests within the NLS were included as inputs and converted to wood products produced from facilities in the NLS. We included wood imports to any of the NLS, and exports from the NLS to anywhere else. Any wood movement from one to either of the other two NLS was treated as retained within the NLS.

Michigan forests cover an estimated 8 million hectares or about 55% of the state’s land area and hold about 1.6 billion metric tons of carbon. The top two species by tree volume in Michigan forests are sugar maple (*Acer saccharum* Marsh.) and red maple (*Acer rubrum* L.) [77]. Minnesota forests cover an estimated 7 million hectares or about 35% of the state’s land area and hold about 1.3 billion metric tons of carbon. The top two species by tree volume in Minnesota forests are quaking aspen (*Populus tremuloides* Michx.) and red pine (*Pinus resinosa* Sol.) [78]. Wisconsin forests cover an estimated 7 million hectares or about 47% of the state’s land area [76] and hold about 1.6 billion metric tons of carbon. The top two species by tree volume in Wisconsin forests are sugar maple (*Acer saccharum* Marsh.) and red maple (*Acer rubrum* L.) [32]. Forest species composition is similar across the NLS, but more so for Michigan and Wisconsin with Minnesota on the western and/or northern range for some northern hardwood species commonly found in the other two NLS. Red maple, sugar maple, yellow birch *(Betula alleghaniensis* Britt.)*,* and eastern hemlock (*Tsuga Canadensis* L.) all have greater densities in Michigan than Minnesota, while Minnesota has greater densities of paper birch *(Betula papyrifera* Marshall)*,* northern red oak *(Quercus rubra* L.)*,* and American basswood (*Tilia americana* L.) [82]. The forests of the NLS have been influenced by large scale clear-cuts and subsequent wildfires during the end of the eighteenth and start of the nineteenth centuries, resulting in more early successional hardwoods than were present pre-European colonization [68]. This compositional shift was further exacerbated by high-grading practices that were common throughout the region [18].

### Harvest estimates

To estimate harvest removals for the NLS for the entire time series 1900–2024, historical and contemporary harvest estimates were needed to supplement the annual NFI data available for the NLS which span the years 2005–2022. To accomplish this, harvests for the CONUS from 1900–2024 that were estimated from the compilation system using wood products estimates from multiple sources (American Forest and Paper Association, American Plywood Association, Bureau of the Census, Composite Panel Association, The Engineered Wood Association, United Nations Food and Agriculture Organization (FAO), USDA Forest Service estimates, USDA Foreign Agricultural Service, USDA Forest Service Timber Product Output (TPO) surveys, US International Trade Commission, and Western Wood Products Association) with years 1990–2024 published in the most recent US GHG inventory [16] were adjusted to address the disparity between these estimates and contemporary removal estimates from the NFI, and then disaggregated to the state-level.

**Fig. 1:**
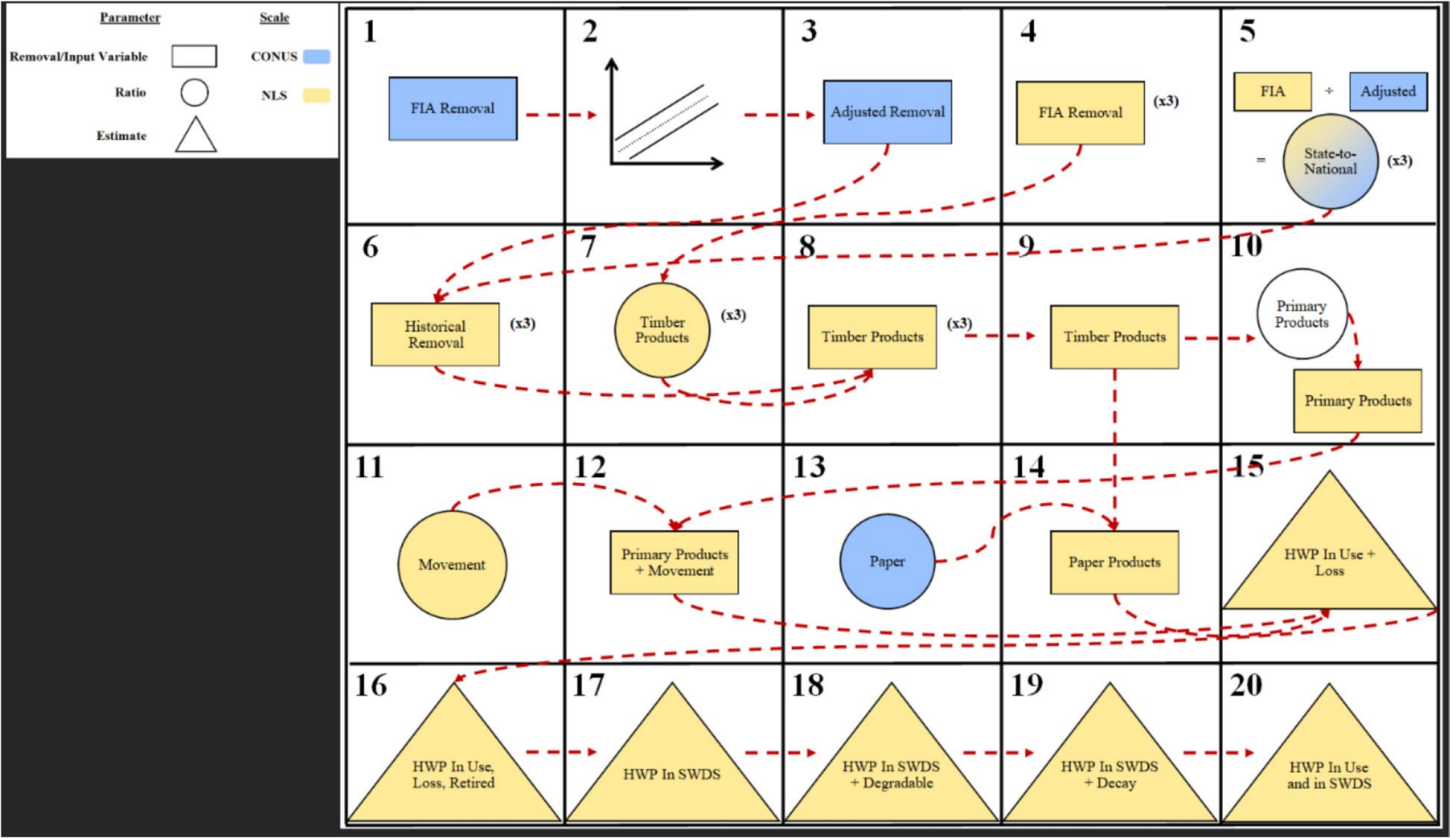
Conceptual flow of methodical steps for compiling estimates of carbon in harvested wood products from nationwide forest inventory (NFI) harvest removal estimates

First, in the data estimation and calibration phase (Steps 1–6, Table 1), the publicly available FIA database (FIADB) was queried to estimate harvest removals for the entire CONUS (see Table 1 and Fig. 1 for step-by-step methodology of the compilation system).

**Table 1:** Methodological steps for compiling estimates of carbon in harvested wood products from nationwide forest inventory (NFI) harvest removal estimates

Step	Description	Data source(s)	Resulting Parameter
(I) 1	Query FIADB for CONUS harvest removals	FIADB 2005–2022	NFI CONUS harvest removals 2005–2022
(I) 2	Perform linear regression on NFI and WOODCARBII CONUS harvest removals from 2005–2022	FIADB 2005–2022; WOODCARBII 2005–2022	Model parameters to adjust historic CONUS harvest
(I) 3	Use model parameters to adjust WOODCARBII harvest estimates for CONUS 1900–2024	WOODCARBII 1900–2024	Adjusted CONUS harvest removals 1900–2024
(I) 4	Query FIADB for NLS harvest removals	FIADB 2005–2022	NFI NLS harvest removals 2005–2022
(I) 5	Divide NLS harvest removal by modeled CONUS harvest removal	FIADB 2005–2022; Adjusted CONUS harvest removals 2005–2022	Mean state-to-national harvest removal ratios for NLS 2005–2022
(I) 6	Multiply mean state-to-national harvest ratios and adjusted CONUS harvests	Mean state-to-national harvest removal ratios 2005–2022; Adjusted CONUS harvests 1900–2024	NLS state-level harvest removal estimates 1900–2024
(II) 7	Calculate state-level timber product ratios (softwood or hardwood, sawlog or pulpwood) from FIADB query in step 4	FIADB 2005–2022	Mean timber product ratios for NLS 2005–2022
(II) 8	Multiply state-level harvest removal estimates and the mean ratio for each timber product in the respective state	NLS state-level harvest removal estimates 1900–2024; Mean timber product ratios for NLS 2005–2022	State-level harvest removal estimates partitioned into different timber products 1900–2024
(II) 9	Sum state-level harvest removal estimates, partitioned into different timber products	State-level harvest removal estimates partitioned into different timber products 1900–2024	Total harvest removal estimates of each timber product in the NLS 1900–2024
(II) 10	Multiply total harvest removal estimates of each timber product in the NLS by primary product ratios from [65] table D6	Total harvest removal estimates of each timber product in the NLS 1900–2024; [65] table D6	Total harvest removal estimates of each primary product (including wood pulp, fuel and other emissions, and bark) in the NLS 1900–2024
(II) 11	Divide the amount of each primary product that is retained in, exported from, and imported to the NLS by the total amount of primary products that were exported or retained (i.e. produced from the region)	TPO 2019	“Movement ratios”—the ratio of each timber product that is retained in, exported from, or imported to the NLS
(II) 12	Multiply total harvest removal estimates of each primary product in the NLS by the ratio of each product that is retained, exported, or imported to the NLS	Total harvest removal estimates of each primary product in the NLS 1900–2024; TPO 2019	Total estimates of primary products retained in, exported from, or imported to the NLS 1900–2024
(II) 13	Divide estimates of each paper product (three values for each product; retained, exported, and imported) by the total pulpwood retained, exported, or imported for the CONUS from WOODCARBII	WOODCARBII 1990–2022	Ratios of each paper product retained, exported, and imported CONUS 1990–2022
(II) 14	Multiply mean paper product ratios by the total amount of pulpwood retained in, exported from, or imported to the NLS	Ratios of each paper product retained, exported, and imported CONUS 1990–2022; Total estimates of primary products retained in, exported from, or imported to the NLS 1900–2024	Paper product estimates for the NLS 1900–2024
(III) 15	Multiply the HWP estimates produced in and exported from the NLS by the fractions of those products that enter end uses in a given year (fractions of sawnwood, structural panels and non-structural panels), and the fractions of products assumed to be lost when places in-use from [62]	Fractions of sawnwood, structural panels, and non-structural panels entering the product in-use pool 1900–2024 [62], Loss in-use 1900–2024 [62]	Estimates of HWPs in-use 1900–2024
(III) 16	Construct arrays for each end use in each year and apply an exponential function to estimate the amount of each product that is retired from use, based on when the product entered the product in-use pool and the half-life of that product	Estimates of HWPs in-use 1900–2024; Half-lives from [62]	Estimates of HWPs in-use and HWPs retired from use 1900–2024
(III) 17	Multiply the estimates of HWPs retired from use by the amount of wood or paper assumed to go to dumps and landfills in a given year	Ratios of wood and paper to dumps and landfills 1900–2024 [62]	Total estimates of HWPs in SWDS 1900–2024
(III) 18	Multiply the estimates of HWPs in SWDS by the ratio of solid wood and paper products subject to decay (degradable stock)	Wood and paper degradable stock in landfills and dumps [62]	Estimates of HWPs in SWDS degradable and non-degradable stock 1900–2024
(III) 19	Construct arrays for each product in each year and apply an exponential function to estimate the amount of each product that decays from SWDS, based on product half-lives and decay rates in SWDS	Half-lives and decay rates in SWDS [62]	Estimates of HWPs in SWDS and decay from SWDS 1900–2024
(IV) 20	Aggregate steps 16–20 into resulting parameters table (see Table 2)		Final HWP Estimates 1900–2024

Steps are grouped into (I): data estimation and calibration, (II): classification of wood products, (III): life-cycle modeling, and (IV): data aggregation

This query included the years 2005–2022 given that 2005 was the first year that allowed for a full estimate of harvest removals from the annual NFI. The FIADB query included harvests of live individual trees ≥ 12.7 cm diameter at breast height (dbh) on all NFI plots that had at least one forest land condition in each state. The aboveground biomass in units of carbon was extracted from the FIADB for harvested trees by multiplying the aboveground biomass of the merchantable bole of each tree by species-specific carbon fractions [81]. Annual estimates of carbon harvested from individual trees on NFI plots were summed in each state to estimate annual state-level harvests.

**Fig. 2:**
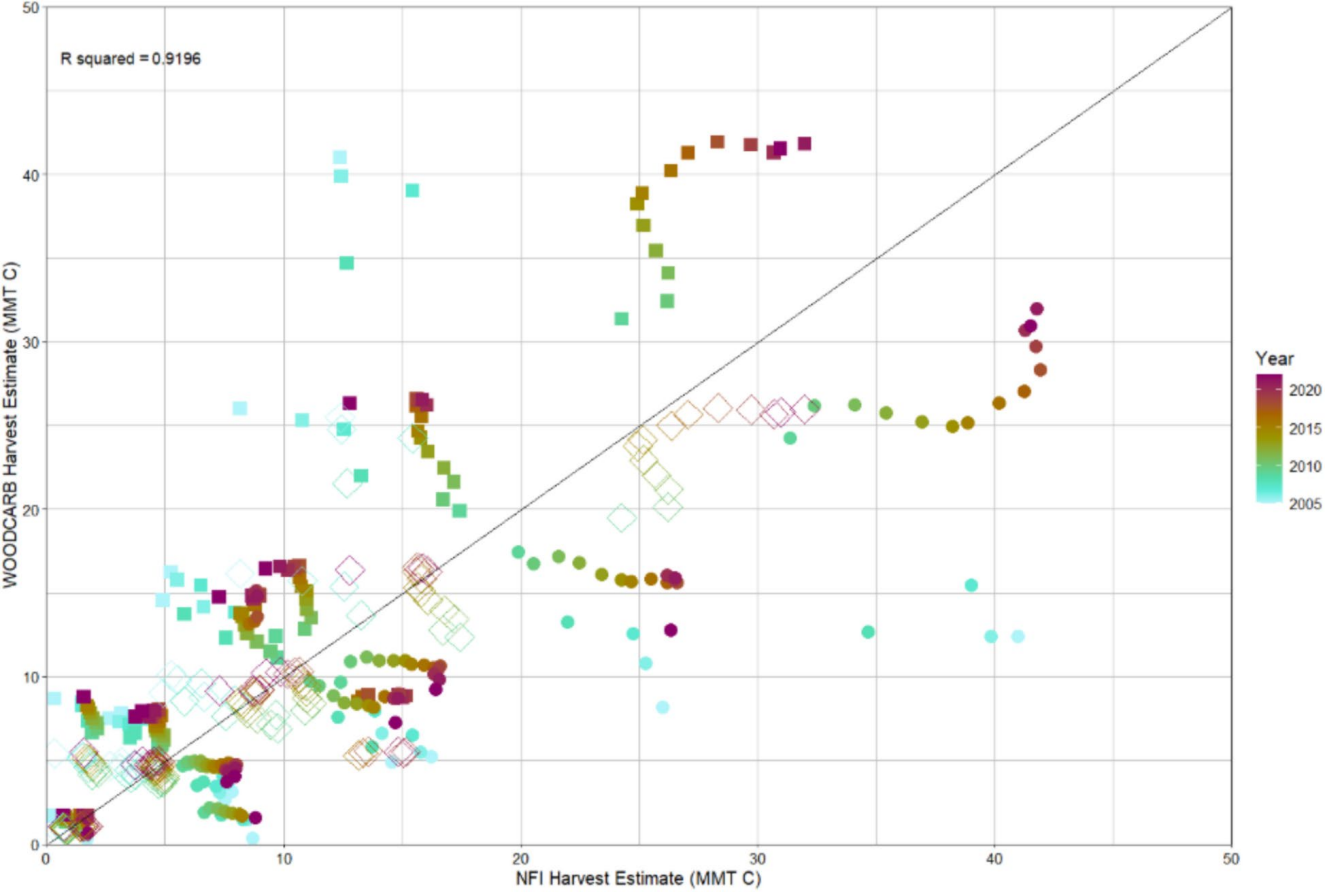
Linear regression between harvest estimates from survey statistics and nationwide forest inventory (NFI) data. Harvest estimates from the WOODCARB model are shown with squares, harvest estimates from NFI data are shown with circles, model predictions are shown with diamonds. The solid black line is a 1:1 line

Next, published [16] harvest estimates were constrained to the same 2005–2022 time series and a simple linear regression model was fit using the two data series, recognizing that the published [16] estimates were consistently greater than those from the FIADB (Fig. 2). Predictions from the linear regression model were used to transform the entire time series from the published [16] harvest estimates.

After obtaining harvest predictions for the CONUS for the entire 1900–2024 time series, the FIADB was queried for harvest removals for the NLS from 2005–2022, the years that allowed for a full estimate from the annual NFI. Logging residues including tops and limbs smaller than merchantable diameter and cull portions of trees are most often left on site following timber harvests in the NLS [13, 38, 46, 83]. To reflect harvesting practices, the estimates of harvest removals from the FIADB are based on the merchantable bole of each harvested tree up to a 10 cm top [85]. The harvest estimate in each state was then divided by the CONUS prediction for the same year to estimate a state-to-national harvest ratio for Michigan, Minnesota, and Wisconsin (0.04 for MI, 0.03 for MN, and 0.04 for WI). The mean state-to-national harvest ratio for each state was then multiplied by the CONUS harvest prediction for each year in the 1900–2024 time series to produce a harvest estimate for each state in the NLS for the entire time series.

The 2005–2022 harvest estimates for the NLS produced from the FIADB query included whether a harvested tree was a softwood or hardwood species, and whether it was of sawlog or pulpwood size based on regional dimensions of harvested trees; 22.9 cm (dbh) for softwoods and 27.9 cm dbh for hardwoods [38, 46, 83]. In the classification of wood products phase (Steps 7–14, Table 1), this information was used to produce timber product ratios (the ratio of each harvest that is pulpwood/sawlog and softwood/hardwood) for each state in the NLS (see additional file 1).

Mean timber product ratios were multiplied by the harvest estimates throughout the entire time series to partition the harvest into various timber product estimates. From this point, the three states were summed to produce harvest estimates, partitioned into timber products, for the entire NLS from 1900–2024.

### US HWP carbon compilation system

Timber products were partitioned into primary products common for country level reporting [31], logs, lumber, plywood, oriented strand board (OSB), non-structural panels, wood pulp, other industrial products, and fuel and other emissions; using northeast regional ratios from [65]. Due to lack of data, to estimate the amount of paper products created from the hardwood and softwood pulpwood as well as the amount of recovered material, the ratio of each paper product to the total pulpwood production in the CONUS from WOODCARBII was calculated for 1990–2022. The mean and standard deviations of each paper product to total pulpwood production was randomly sampled 10,000 times assuming a normal distribution, and the mean of those sample runs was applied to estimate pulpwood production in the NLS to produce estimates for each paper product.

The amounts of each primary product that were retained in, exported from, and imported to the NLS from the most recent complete TPO data for the region (2019) were divided by the total amount of primary products that were exported or retained (i.e., produced from the region) to calculate the ratios of each product that would be allocated to these movement categories (see additional file 1).

In the life-cycle modeling phase (Steps 15–19, Table 1), ratios from Skog [62] were then used to allocate carbon in solidwood primary products to 13 different end uses including single-family housing, multifamily housing, residential upkeep and improvement, and other uses – which included mobile homes, non-residential construction, rail ties, rail cars, household furniture, commercial furniture, other manufacturing, shipping, and miscellaneous other. One category was used for all paper and paperboard uses. An 8% loss following McKeever [35] and Skog [62] was assumed for the starting amount of products to account for waste, i.e., products that are discarded before entering the in-use pool. First order (exponential) decay models with half-live estimates (e.g., the time at which half of the amount of products in-use will have been discarded from use) from Skog [62] were used to predict how long products stayed in-use and the rate of decay in SWDS. The US HWP CCS then aggregates the data (Step 20, Table 1) to produce the final estimates.

### Uncertainty and assumptions

The US HWP CCS can produce estimates down to any level with sufficient harvest data, but due to a lack of data on local ratios, half-lives, and decay rates national scale data are disaggregated with the assumption that these estimates are representative of state or entity-level parameters. Regional primary product ratios from [65] were used to represent the ratio of primary products in the NLS. Timber products output data from the 2019 mill surveys were used to obtain ratios of each product that is retained in, exported from, and imported to the NLS throughout the entire time series. Additionally, a ratio of paper products to total pulpwood on a national scale was used for the NLS.

Sources of uncertainty were integrated into the compilation system by bootstrapping from the available data for each ratio. Furthermore, the national fractions of products placed into various end uses, the half-lives of those products, their loss when placed in-use, the proportions sent to solid waste disposal sites, and the decay in solid waste disposal sites from Skog [62] were used to represent those parameters in the NLS for the entire time series.

**Fig. 3.**
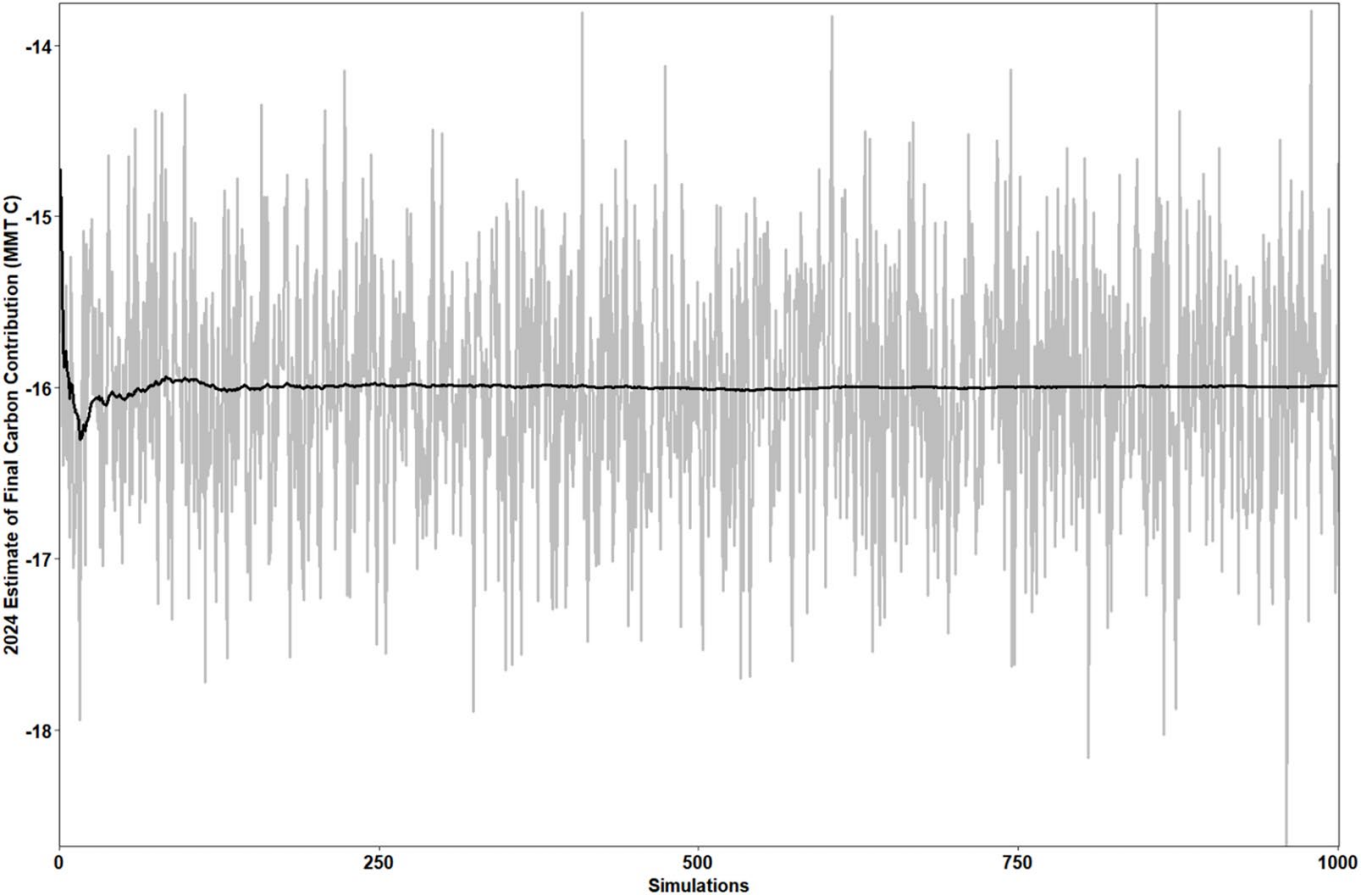
2024 Estimate of final carbon contribution (MMT C) calculated using the production approach for the Northern Lakes States (MI, MN, WI), US. Gray lines represent the individual estimates across 1,000 random simulations, and the black line represents the mean across simulations

Available input data was bootstrapped to characterize uncertainty. The ratios used to produce harvest removals, timber product estimates (from FIADB query of years 2005–2022), primary product estimates (from [65] ± 15% uncertainty with an assumed normal distribution), and paper estimates (from ratios of each paper product to pulpwood production, imports, and exports in original compilation process 1990-2022, [86] were randomly sampled from the mean and standard deviations of the available data rather than using one static estimate. The HWP CCS was run 1,000 times drawing randomly from the distributions of the available input data and convergence (where the difference in means when incorporating additional individual estimates did not deviate significantly from zero) was obtained after approximately 200 runs (Fig. 3).

The range of data for each of these parameters was then propagated through the compilation system to produce a distribution for each end parameter, rather than a single estimate.

### Sensitivity analysis

We conducted a sensitivity analysis to determine the contribution of various inputs to the final outputs of the modeling framework. To conduct the sensitivity analysis, we did a step-wise transformation where one input variable was altered at a time by an amount large enough to produce a noticeable change in the outputs. We chose a 50% increase for each input variable while holding all other input variables constant and ran the compilation system. We iterated this method for each variable to determine the impact of a 50% increase on the results of the compilation system.

Harvest estimates were queried, data was compiled and simulated, and figures were created in R [58].

## Results

### Annual harvest estimates

**Fig. 4:**
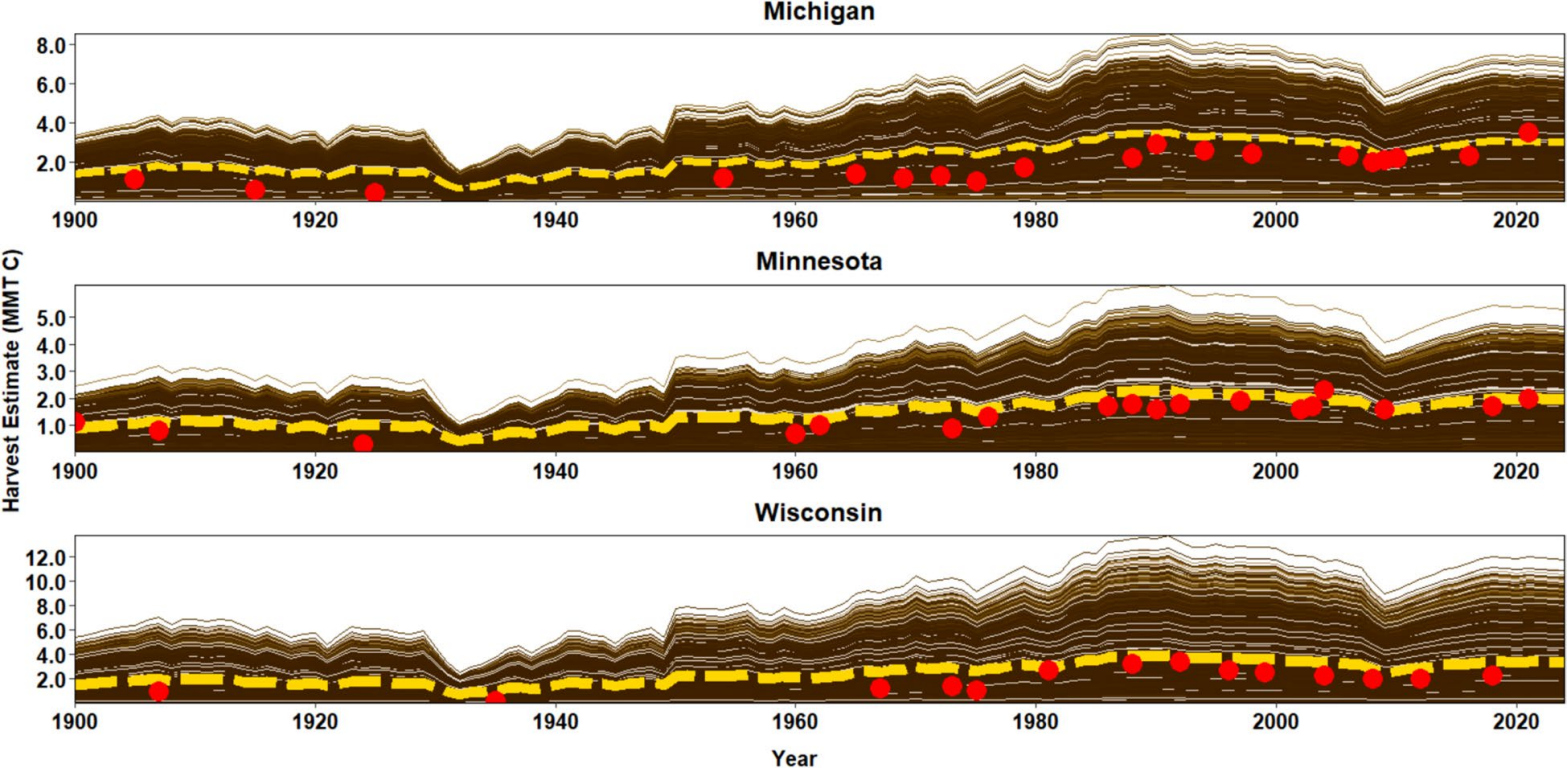
Annual harvest estimates from 1900–2024 for the Northern Lake States, US (MI, MN, WI). Brown lines are each of 10,000 random samples from a normal distribution of the state to national harvest ratio, yellow dashed line is the mean of all the random samples, and red dots are harvest estimates from the literature

Estimates of the annual harvest from the NLS were lowest in the 1930s, with the smallest harvest in 1932 of 1.7 million metric tons of carbon (MMT C) ± 0.03 MMT C (uncertainty is standard error unless specified otherwise) (Fig. 4).

While individual annual harvest estimates fluctuated, the overall pattern was an increase in annual harvests after 1932 to a peak of 9.2 MMT C ± 0.2 MMT C in 1991, followed by a gradual decline. Annual harvests quickly declined from 2008 to 2011, before again gradually increasing to about 8.0 MMT C throughout the late 2010s and 2020s. We estimate a 2024 harvest of 3.0 ± 0.01 MMT C, or 0.6% of the total C in forest aboveground biomass (AGB) in Michigan, 2.0 ± 0.01 MMT C or 0.6 % of the total C in AGB in Minnesota, and 3.4 ± 0.02 MMT C or 0.8% of the total C in AGB in Wisconsin, with a total annual harvest of 8.3 ± 0.04 MMT C for the entire NLS. Our state-level estimates of annual harvested carbon were 0.04 ± 0.01% (standard deviation) of the national harvest estimate from the FIADB for Michigan, 0.03 ± 0.01% (standard deviation) for Minnesota, and 0.04 ± 0.03% (standard deviation) for Wisconsin. Our mean state-level harvest estimates were generally slightly higher than reported estimates from the literature for all three of the NLS (MI: [3, 4, 10, 23, 24, 29, 50, 54–57, 63, 77], MN: [6, 21, 22, 27, 39–44, 47, 59, 67, 73, 74, 78, 79], WI: [5, 7, 25, 26, 28, 33, 51–53, 60, 64, 66, 73, 75].

**Fig. 5:**
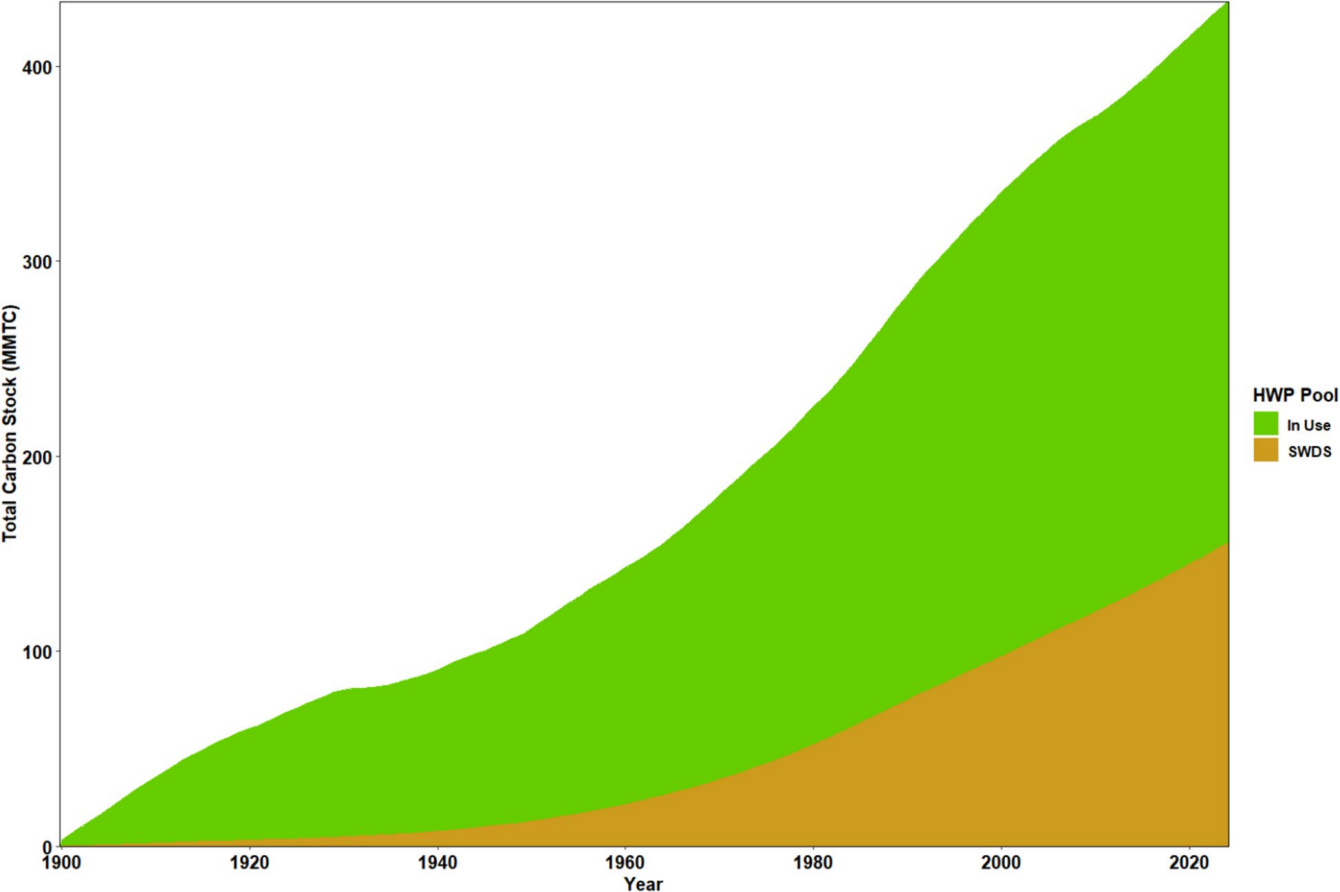
Cumulative carbon stock (MMT C) for harvested wood products in-use and in solid waste disposal sites (SWDS) for the Northern Lake States, US (MI, MN, WI) calculated using the IPCC production approach

**Fig. 6:**
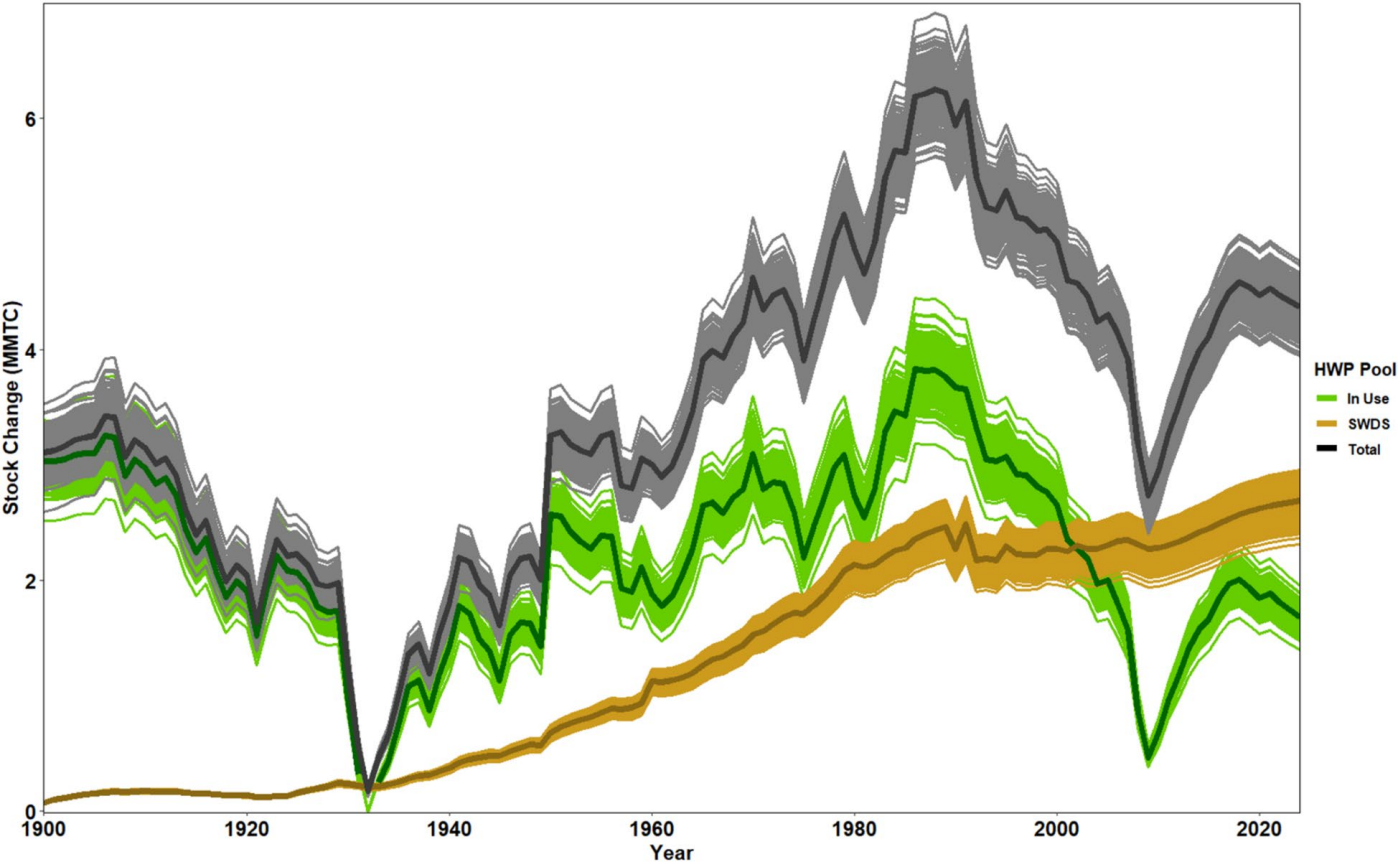
Annual stock change (MMT C) for harvested wood products (HWPs) in-use and in solid waste disposal sites (SWDS) for the Northern Lake States, US (MI, MN, WI) estimated using the IPCC production approach. Ranges for each estimate were produced from 250 independent runs of the compilation system, means are shown as solid dark lines

Estimates for years more recent than 2000 were more closely aligned with estimates from the literature than those from 1900–1990 (Fig. 4). Our harvest estimates for years since 2000, however, were not identical to the FIA state-level reports of harvested removals due to the calibration of our harvest estimates with survey statistics data. Harvest estimates are often reported in cubic feet and therefore in order to compare our estimates in units of carbon to those reported in the literature we assumed a conversion of 14.90 thousand cubic feet to oven dry tons and 4.54e-07 oven dry tons to MMT C [62]. Carbon in HWPs from the NLS under the production approach represented an estimated total accumulation of 277.0 ± 17.5 MMT C stored in solidwood and paper products in-use and 155.2 ± 9.8 MMT C in SWDS from 1900–2024 (Fig. 5) with a carbon sink of 4.9 ± 0.1 MMT C in 2024 (Fig. 6).

This includes HWPs produced in the NLS and exported domestically or internationally, as well as any HWPs produced and retained in the NLS, but not imports. The annual input of carbon to products in-use was variable, with the largest input in 1991 (8.6 MMT C) and the lowest in 1932 (1.6 MMT C). Estimates of cumulative carbon in HWPs increased throughout the time series, peaking at 489.1 MMT C in 2024, with 313.6 MMT C in-use and 175.5 MMT C in SWDS (Fig. 5). In 2024, an estimated 7.3 ± 0.2 MMT C were added to the HWPs in-use and 3.1 ± 0.1 MMT C in SWDS.

The largest estimated HWP imports (0.3 MMT C) and exports (0.2 MMT C) in the NLS were in 1991, while the smallest annual imports (0.05 MMT C) and exports (0.04 MMT C) were in 1932. In 2024, an estimated 0.3 MMT C or 4% of the total annual HWP stock were imported and 0.2 MMT C or 3% of the total annual HWP stock were exported, with 93% of HWPs retained within the three NLS (Table 2).

**Table 2:** Harvest wood product (HWP) estimates for the Northern Lake States, US (MI, MN, WI), 2000–2024 calculated using the IPCC Production approach

Inventory year	Annual Change in stock of HWP in-use from consumption	Annual Change in stock of HWP in SWDS from consumption	Annual Change in stock of HWP in-use produced from domestic harvest	Annual Change in stock of HWP in SWDS produced from domestic harvest	Annual Imports of wood, and paper products + wood fuel, pulp, recovered paper, roundwood/ chips	Annual Exports of wood, and paper products + wood fuel, pulp, recovered paper, roundwood/ chips	Annual Domestic Harvest
	**MMT C/yr**						
2000	3.01	2.56	3.01	2.55	0.28	0.22	8.50
2001	2.66	2.54	2.66	2.53	0.27	0.21	8.13
2002	2.57	2.60	2.57	2.59	0.26	0.20	8.06
2003	2.48	2.56	2.48	2.55	0.26	0.20	8.04
2004	2.23	2.56	2.23	2.55	0.25	0.20	7.74
2005	2.27	2.59	2.27	2.58	0.26	0.20	7.85
2006	2.04	2.63	2.04	2.62	0.25	0.19	7.63
2007	1.77	2.64	1.77	2.63	0.25	0.19	7.47
2008	0.99	2.60	0.99	2.59	0.22	0.17	6.64
2009	0.52	2.55	0.53	2.55	0.20	0.15	6.01
2010	0.77	2.56	0.77	2.56	0.20	0.16	6.20
2011	1.09	2.59	1.09	2.59	0.21	0.17	6.53
2012	1.34	2.63	1.34	2.62	0.22	0.17	6.78
2013	1.60	2.67	1.59	2.66	0.23	0.18	7.07
2014	1.79	2.72	1.78	2.71	0.24	0.19	7.32
2015	1.89	2.76	1.89	2.75	0.24	0.19	7.44
2016	2.09	2.81	2.09	2.80	0.25	0.20	7.70
2017	2.23	2.85	2.23	2.84	0.26	0.20	7.90
2018	2.28	2.90	2.28	2.89	0.26	0.20	8.03
2019	2.19	2.93	2.19	2.92	0.26	0.20	7.99
2020	2.10	2.95	2.09	2.94	0.26	0.20	7.91
2021	2.14	2.98	2.13	2.97	0.26	0.20	8.00
2022	2.05	3.00	2.05	2.99	0.26	0.20	7.95
2023	1.97	3.01	1.97	3.00	0.26	0.20	7.89
2024	1.91	3.03	1.90	3.02	0.26	0.20	7.84

**Fig. 7:**
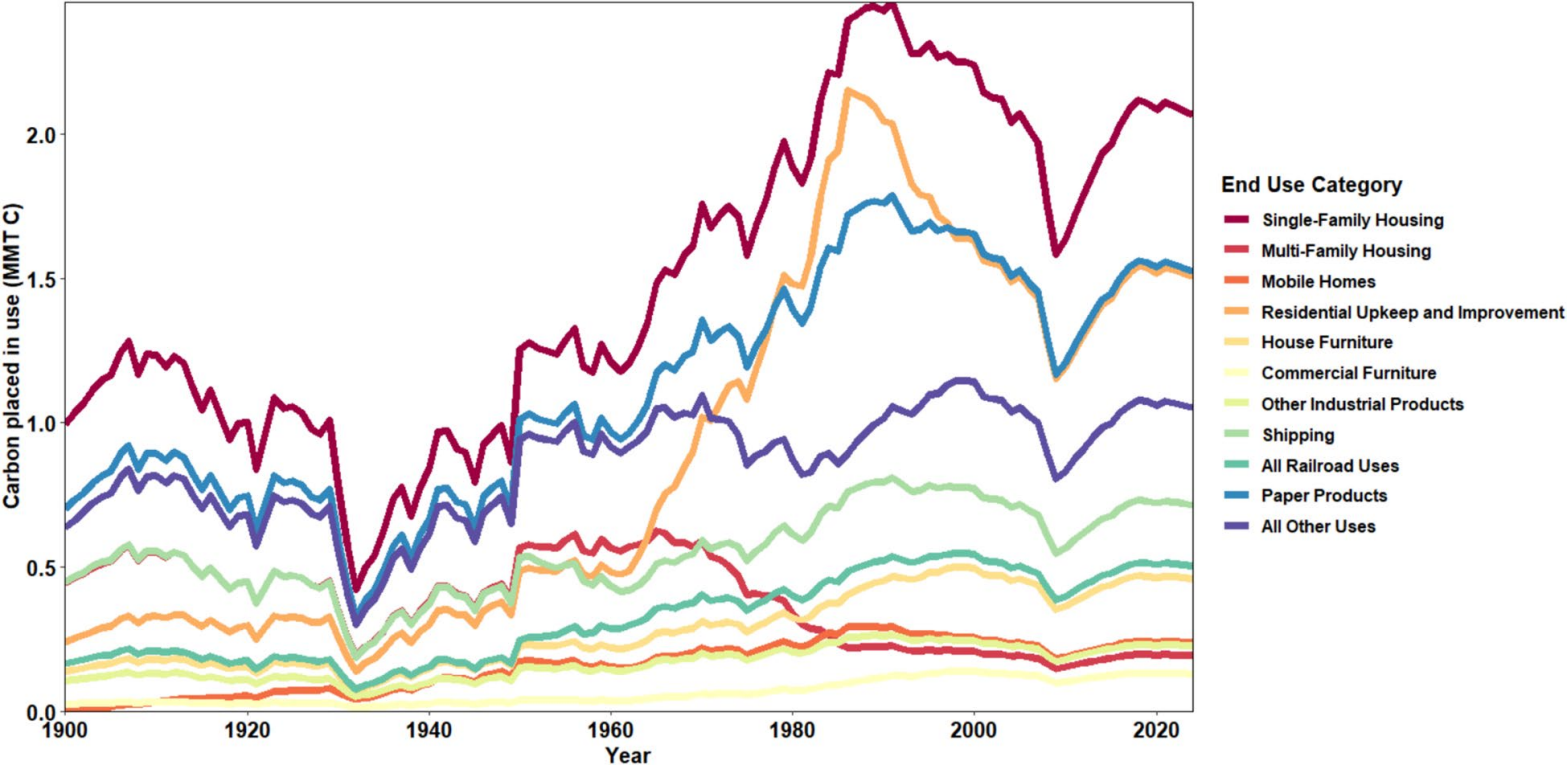
Carbon placed in various end uses (MMT C/year) from 1900–2024 for the Northern Lake States, US (MI, MN, WI)

Of the end uses, the largest annual inputs of carbon were to single-family housing, followed by residential upkeep and improvement (Fig. 7).

There were two periods where inputs were particularly low to the HWPs in-use pool, especially inputs to single-family housing, multi-family housing, and residential upkeep and improvements: from 1931–1935 and 2008–2010 (Fig. 7).

The annual change in carbon from products in-use was more variable than that of products in SWDS, which has been gradually increasing since the 1900s (Fig. 6). Due to assumptions that the product uses, half-lives, and decay rates are the same whether the HWPs are exported or retained, the above pattern held true for retained and exported products. An estimated 3.0 ± 0.07 MMT C burned as fuelwood in the NLS in 2024. Throughout the 1900–2024 time series mean annual estimates of fuelwood ranged from 0.6 MMT C in 1932 to 3.5 MMT C in 1991.

### Sensitivity analysis

**Fig. 8:**
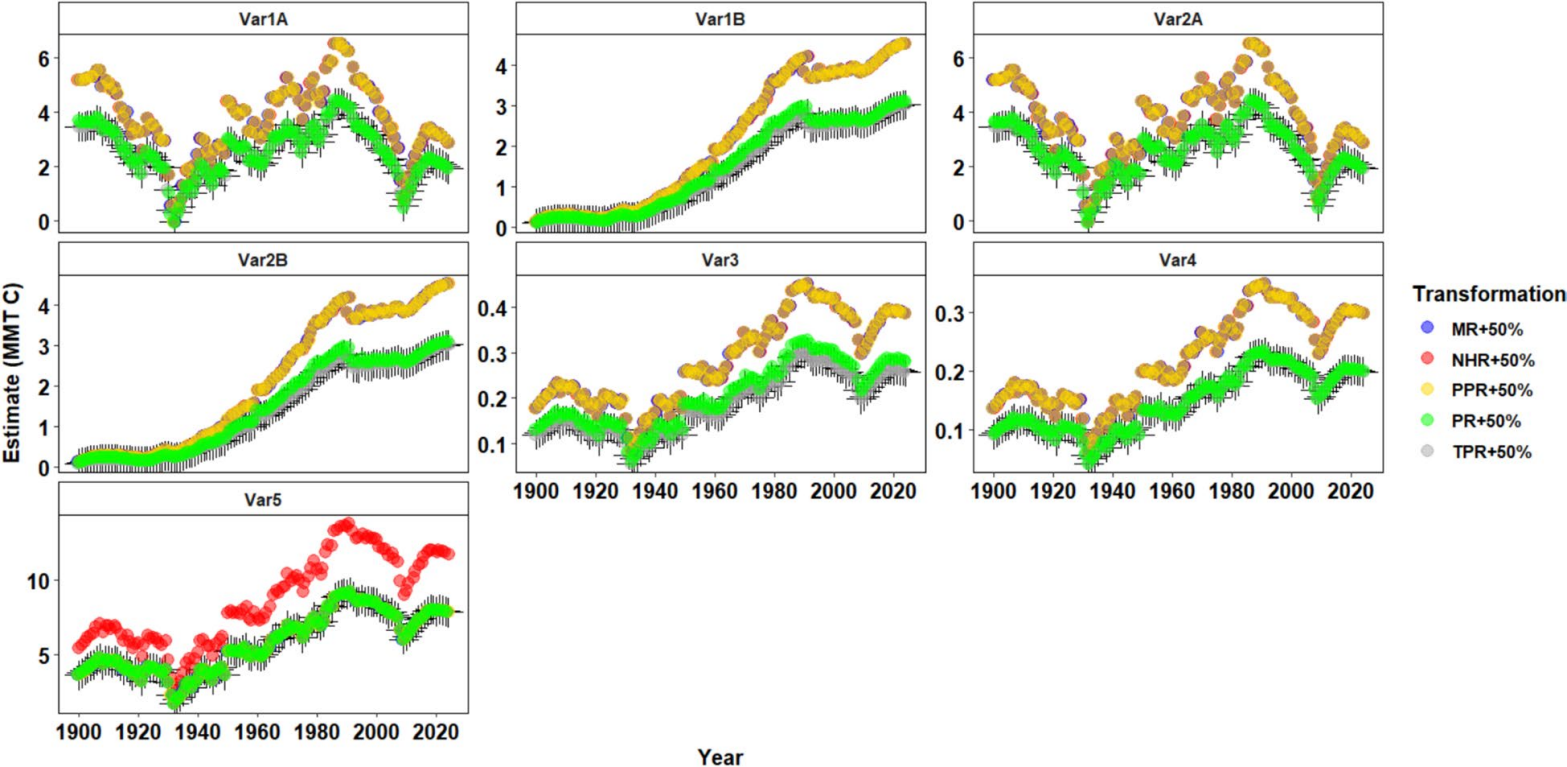
Sensitivity analysis of final IPCC variables (MMT C) calculated using the production approach from 1900–2024 for the Northern Lake States, US (MI, MN, WI). Variables 1A-5 represent the annual change in stock of HWP in-use from consumption, the annual change in stock of HWP in solid waste disposal sites (SWDS) from consumption, the annual change in stock of HWP in-use produced from domestic harvest, the annual change in stock of HWP in SWDS from domestic harvest, the annual imports of wood and paper products, the annual exports of wood and paper products, and the annual domestic harvest, respectively. Transformations represent sensitivity analyses to determine the impact of altered input ratios on the final results. MR = movement ratio, the amount of products imported, exported, or retained. NHR = national harvest ratio, the ratio of each state’s harvest to the national US harvest. PPR = primary product ratios, the ratios of each primary timber product. PR = paper ratios, the ratios of each paper product to the total pulpwood. TPR = timber product ratios, the ratio of softwood and pulpwood to total roundwood removed from the forest

Sensitivity analysis revealed that of the input ratios, the movement ratios (the amount of products imported, exported, or retained), the state to national harvest ratios, and the primary product ratios, respectively, have the largest influence on the final results of the compilation system (Fig. 8).

The resulting mean change in the annual carbon contribution from a 50% increase in each ratio was a 37.1% increase in stored carbon for the state to national harvest ratios, a 7.5% decrease in stored carbon for the removal ratios, a 38.2% increase in stored carbon for the movement ratios, a 43.6% increase in stored carbon for the primary product ratios, and a 3.6% decrease in stored carbon for the paper ratios. A 50% increase in the fractions of structural panels, non-structural panels, and sawnwood resulted in 3.2%, 0.6%, and 27.0% increases of annual carbon contribution, respectively. A 50% increase in all of the half-lives for products in end uses resulted in a 1.1% increase in the annual carbon contribution. The half-lives that were most influential on the annual carbon contribution were for products in single-family housing, residential upkeep and improvement, and shipping, followed by furniture manufacturing, multi-family housing, mobile homes, and then all other uses. Altering the decay rates of solidwood and paper products in SWDS and the non-degradable portion of materials in landfills changed the rate of accumulation but did not alter the total carbon output of SWDS.

## Discussion

The advances documented in this case study through the development of the US HWP CCS provide an avenue to disaggregate national estimates of carbon in HWPs to the state and entity level. Incorporating NFI and TPO data directly into the new compilation system eliminated several assumptions that were previously necessary in the HWP carbon estimation process. First, rather than using a generic 50% carbon fraction for all harvested wood, species-specific carbon fractions were used following the most contemporary national-scale volume and biomass (NSVB) methods in the FIADB [81]. Furthermore, rather than assuming a series of conversions to estimate the biomass of primary products from timber product volumes, timber product estimates were already in units of carbon from the public-facing FIADB – which also allowed for estimation of softwood and hardwood sawlogs and pulpwood based on individual tree species and size. Harvest removals were therefore converted into primary products and end uses using only 6 sets of ratios (i.e., state-to-national harvest ratios, timber product ratios, primary product ratios, movement ratios, paper ratios, end use ratios), eliminating the need for 21 distinct unit conversions from industry standard units and two carbon content conversions (for solidwood and paper products) in the previous process. Although the US HWP CCS allows for any method of HWP accounting, the results reported in this case study follow the production method and it was assumed that all HWPs exported follow the same half-lives when in-use and decay rates in SWDS in their destination as they would in the NLS. These assumptions can be eliminated with the integration of distinct half-lives and decay rates for exported products if they are available or developed. Distinct disposition pathways could also be incorporated for regional specificity, and the relative proportions of products disposed, burned, and recycled alters the climate warming impact of HWPs [71]. The assumptions related to solidwood and paper product ratios and the fractions of sawnwood, structural panels, and non-structural panels could also be eliminated with the integration of more specific, localized data.

Although the HWP CCS was developed for use with US NFI data it can be run using any estimate of harvested carbon as a starting point. A robust forest inventory and species-specific carbon fractions such as those incorporated into the US NFI allow for increased accuracy and precision of estimates, however, the system can be used to estimate carbon in HWPs from estimates of harvested carbon at any scale.

The incorporation of NFI data into the US HWP CCS also more closely aligns estimates of carbon transferred from the forest ecosystem into HWPs, where NFI data were used for forest ecosystem carbon estimation. In the past, HWP carbon estimation of solidwood and paper products were compiled from a variety of external sources, namely survey statistics from HWP producing facilities [16] and the compilation process did not include uncertainties associated with these data sources. The input data to the HWP CCS use a distribution informed by the most contemporary NFI and TPO data available to incorporate uncertainty into the compilation system rather than simply using the survey estimates themselves. The use of state-to-national harvest ratios, timber product ratios, and movement ratios informed by NFI and TPO data also eliminated some dependence on external data sources, which are not always available at small spatial scales (i.e. state and sub-state). The flexibility in the HWP CCS, however, allows for the integration of external data sources provided that they are available on the intended scale and more contemporary than alternative NFI and TPO data.

In some cases, estimates of HWPs from TPO survey statistics are more contemporary than estimates of harvested trees from NFI plots due to the annualization of the surveys. While TPO survey statistics can sometimes be more contemporary, their use in HWP carbon estimation created a disparity between estimates of carbon in forest ecosystem pools in the US, namely aboveground biomass, and estimates of carbon in HWPs. The harmonization of NFI and TPO data from the FIA program reduces this disparity and provides estimates that are more consistent with those in forest ecosystem carbon pools compiled using NFI data.

The magnitude and trend of estimates of carbon in HWPs can be verified by relating the estimates to major economic events that influence wood production and consumption. For example, inputs to the HWP in-use pool were particularly low from 1931–1935 and from 2008–2010, reflecting economic downturns as a result of the great depression and the great recession [15]. These economic events are apparent in the amount of HWPs entering the in-use pool on a slight lag – i.e., the decrease in HWPs entering the in-use pool occurs 1–2 years after the economic downturn began. This lag is most likely driven by the time required to compile inventory data and for the time it takes for wood processing facilities to see the impacts of reduced wood consumption.

Our estimates from this pilot study in the NLS are about 20% of the national estimate produced from the original compilation system for the US for carbon in HWPs in-use and about 14% of the national estimate in SWDS [14]. Our harvest estimates were closest to the estimates reported in Minnesota [78], and furthest in Wisconsin [76]. Our 2021 harvest estimate was 3.1 MMT C in Michigan, compared to 3.7 MMT C reported by the FIA program [77]. In Minnesota, our 2021 harvest estimate was 2.0 MMT C, which was the same as the USDA Forest Service FIA estimate [78]. In Wisconsin, our 2018 harvest estimate was 3.5 MMT C, compared to the 2.3 MMT C reported by the USDA Forest Service FIA program [76].

Finally, the sensitivity analysis in this case study informs which inputs warrant the most immediate attention and investment to reduce uncertainty and increase the precision of our estimates at the scale of the NLS. By design, the new HWPs CCS eliminates the need for factors to convert wood products to carbon, which were among the variables that were most influential to the final estimate of HWP carbon in the original framework [86]. Beyond carbon fractions, the sensitivity analysis in this case study suggests that carbon estimates of HWPs are most sensitive to changes in primary product ratios and fractions of sawnwood in various end uses. These findings are consistent with those from [86] and the results from the sensitivity analysis provide a quantitative approach for prioritizing future investments in data acquisition and/or research in support of more contemporary and disaggregated estimates for these key variables.

## Conclusions

This case study documents the new HWP CCS and improvements that enable users to disaggregate national estimates of carbon in HWPs to state and sub-state levels. The compilation system reduces several assumptions that were required in the previous compilation process to produce estimates such as unit conversions and carbon content assumptions. We have integrated data from the NFI that includes species-specific carbon fractions and constrains harvest estimates to more closely align estimates of carbon in HWPs with estimates of carbon in forest ecosystem pools.

While estimates of carbon in HWPs in-use and in SWDS remain uncertain, the new HWP CCS facilitates the quantification and propagation of uncertainties in several key inputs. Furthermore, the results from the sensitivity analysis provide a quantitative approach to prioritize future investment to improve data inputs and reduce uncertainty. This updated compilation system provides an avenue for national, state, and entity-level estimates of carbon in HWPs in-use and in SWDS with minimal data (Tier 1 or Tier 2), and also allows for integration of specific, regional data (Tier 3) while ensuring that estimates at any scale more closely align with estimates of forest ecosystem carbon stocks and stock changes obtained from NFI data.

## Supplementary Information

Supplementary material 1.

## Data Availability

The NFI data are available through the USDA Forest Service FIA DataMart (https://research.fs.usda.gov/products/dataandtools/fia-datamart). The R package is available on request.
