# Supplementary material for: Advancing carbon estimation in harvested wood products in the United States: a case study in the Northern Lake States
Source: Carbon Balance Manag. 2026 Jun 8;21:103. doi: 10.1186/s13021-026-00464-y (PMC13452093; doi:10.1186/s13021-026-00464-y)
Supplement: Supplementary file 1 [file 13021_2026_464_MOESM1_ESM.docx]

Ratios used to partition harvests into various timber products for the Northern Lake States, US (MI, MN, WI).

|  | | MI | MN | WI |
| --- | --- | --- | --- | --- |
| Soft Hardwoods Pulpwood | Mean | 0.14 | 0.30 | 0.21 |
|  | Standard deviation | 0.01 | 0.03 | 0.01 |
| Soft Hardwoods Sawlog | Mean | 0.23 | 0.37 | 0.22 |
|  | Standard deviation | 0.02 | 0.01 | 0.01 |
| Hard Hardwoods Pulpwood | Mean | 0.11 | 0.03 | 0.11 |
|  | Standard deviation | 0.01 | 0.01 | 0.004 |
| Hard Hardwoods Sawlog | Mean | 0.31 | 0.07 | 0.27 |
|  | Standard deviation | 0.02 | 0.01 | 0.03 |
| Pines Pulpwood | Mean | 0.04 | 0.04 | 0.04 |
|  | Standard deviation | 0.01 | 0.01 | 0.01 |
| Pines Sawlog | Mean | 0.09 | 0.07 | 0.10 |
|  | Standard deviation | 0.03 | 0.01 | 0.02 |
| Other Softwoods Pulpwood | Mean | 0.03 | 0.07 | 0.02 |
|  | Standard deviation | 0.003 | 0.01 | 0.003 |
| Other Softwoods Sawlog | Mean | 0.05 | 0.05 | 0.02 |
|  | Standard deviation | 0.01 | 0.01 | 0.01 |

Ratios of products exported, imported, or retained for the Northern Lake States, US (MI, MN, WI).

|  | | **Logs** | **Lumber** | **Plywood** | **OSB** | **NSP** | **Other Industrial Products** | **Wood Pulp** | **Fuel** |
| --- | --- | --- | --- | --- | --- | --- | --- | --- | --- |
| **Exports** | **Softwood** | 0.000 | 0.000 | 0.000 | 0.000 | 0.000 | 0.003 | 0.000 | 0.013 |
|  | **Hardwood** | 0.000 | 0.102 | 0.000 | 0.000 | 0.000 | 0.008 | 0.001 | 0.000 |
| **Imports** | **Softwood** | 0.021 | 0.057 | 0.000 | 0.000 | 0.000 | 0.000 | 0.021 | 0.000 |
|  | **Hardwood** | 0.000 | 0.092 | 0.014 | 0.014 | 0.014 | 0.003 | 0.025 | 0.001 |
| **Retained** | **Softwood** | 1.000 | 6.000 | 1.000 | 1.000 | 1.000 | 0.997 | 1.000 | 0.987 |
|  | **Hardwood** | 0.000 | 1.898 | 1.000 | 1.000 | 1.000 | 0.992 | 0.999 | 1.000 |
